# Correlation of alternative healthy eating index with risk of frailty among metabolic syndrome individuals: a cross-sectional study

**DOI:** 10.1007/s40520-025-02992-y

**Published:** 2025-03-17

**Authors:** Yi Wei, Min Zha, Jiangyi Yu

**Affiliations:** Department of Endocrinology, Jiangsu Province Hospital of Chinese Medicine, Affiliated Hospital of Nanjing University of Chinese Medicine, Nanjing, China

**Keywords:** Alternative healthy eating index, Metabolic syndrome, frailty, NHANES, cross-sectional study

## Abstract

**Background:**

Mounting evidence identifies diet quality as a frailty modifying factor. Individuals suffering from metabolic syndrome (MetS) are more likely to be affected by frailty. Therefore, our research sought to explore the relationship of Alternative Healthy Eating Index (AHEI) with frailty risk among patients with MetS.

**Methods:**

National Health and Nutrition Examination Survey (NHANES) data from 2005 to 2018 were gathered. Frailty Index (FI) was utilized for assessment of frailty status. Weighted multivariate logistic regression model was adopted for investigating the association of AHEI with frailty among patients with MetS. Subgroup analysis, interaction test and restricted cubic spline (RCS) test were also performed in this study.

**Results:**

When the covariates considered were entirely adjusted for, higher AHEI scores exhibited significant association with reduced frailty risk (OR = 0.99,95%CI = 0.981–0.998, *P* = 0.022). Relative to the frailty risk among participants belonging to the lowest AHEI quartile(Q1), that of individuals in the highest AHEI quartile(Q4) decreased by 32% (OR = 0.68, 95% CI = 0.51–0.92, *P* = 0.01). Additionally, the negative association of AHEI with frailty persisted for all subgroup analyses, which also indicates the reliability of the relationship.

**Conclusion:**

For patients with MetS, higher AHEI scores reduce the risk of developing frailty. This investigation provides valuable knowledge that could be utilized for treating MetS patients clinically and guiding healthy eating program development.

**Supplementary Information:**

The online version contains supplementary material available at 10.1007/s40520-025-02992-y.

## Introduction

Metabolic syndrome (MetS) refers to metabolic abnormalities exhibiting presentations of central adiposity, dyslipidemia, hypertension, and hyperglycemia [1]. Since Western lifestyles have increasingly been adopted by people around the world, MetS incidence has recently risen dramatically to alarming levels and the disease is now a truly global problem [2]. In North America, particularly the United States, the overall prevalence of MetS increased from 37.6% in the 2011–2012 cycle to 41.8% in the 2017–2018 cycle according to the National Health and Nutrition Examination Survey (NHANES) survey [3]. Among Chinese individuals with an age of ≥ 15 years, the pooled prevalence of MetS was 24.5%, according to a meta-analysis [4]. In addition, MetS also displayed relationships with markedly elevated incidence of mortality caused by diabetes mellitus (DM), heart disease and any other reasons [5].

MetS individuals are more likely to develop frailty. A previous study on Chinese individuals aged ≥ 45 years revealed that MetS severity was linked with frailty advancement [6]. Another investigation revealed that older persons with MetS exhibited a higher propensity for frailty compared to their counterparts without MetS [7]. In addition to the evident correlation between MetS and frailty, a previous quantitative systematic review also exhibited that MetS was significantly correlated with decreased body weight, reduced speed of gait, and impaired physical activity [8]. Therefore, screening for frailty in population with MetS is important and has strong value in clinical practice.

Nutrients are typically ingested not in isolation, but within meal groups comprising many foods, which is regarded as a contributing element to the presence of synergy in intricate food combinations [9]. The Alternative Healthy Eating Index (AHEI)-2010 is an effective instrument for assessing compliance with dietary guidelines and evidence-based recommendations. Higher AHEI scores strongly indicated decreased incidence of common chronic disorders, such as DM and heart and blood vessel diseases [10–14]. A Luxembourg research team cross-sectionally analyzed the AHEI-MetS association and identified negative correlations between the AHEI and components of MetS, comprising fasting plasma glucose (FPG), body mass index (BMI), waist circumference (WC), and blood pressure [15]. A study conducted by a research team in Iran found that for overweight/obese adolescents, following the AHEI dietary pattern reduces the prevalence of metabolically unhealthy overweight/obesity (MUO) [16]. Reeder et al. found the beneficial effects of high-AHEI-score diet on reducing MetS development risk in an African American cohort [17]. However, whether AHEI is implicated with frailty development risk among individuals affected by MetS remains elusive.

Therefore, this research utilized NHANES data to explore this issue in a cross-sectional study. Our findings are presented herein with the intention of enhancing the clinical management of patients who have metabolic syndrome, effectively preventing the development of frailty, and providing useful advice on the dietary habits of these patients.

## Methods

### Data source and cohort collection

Data of seven NHANES cycles from 2005 to 2018, which can be freely downloaded from https://www.cdc.gov/nchs/nhanes/.

**Fig. 1:**
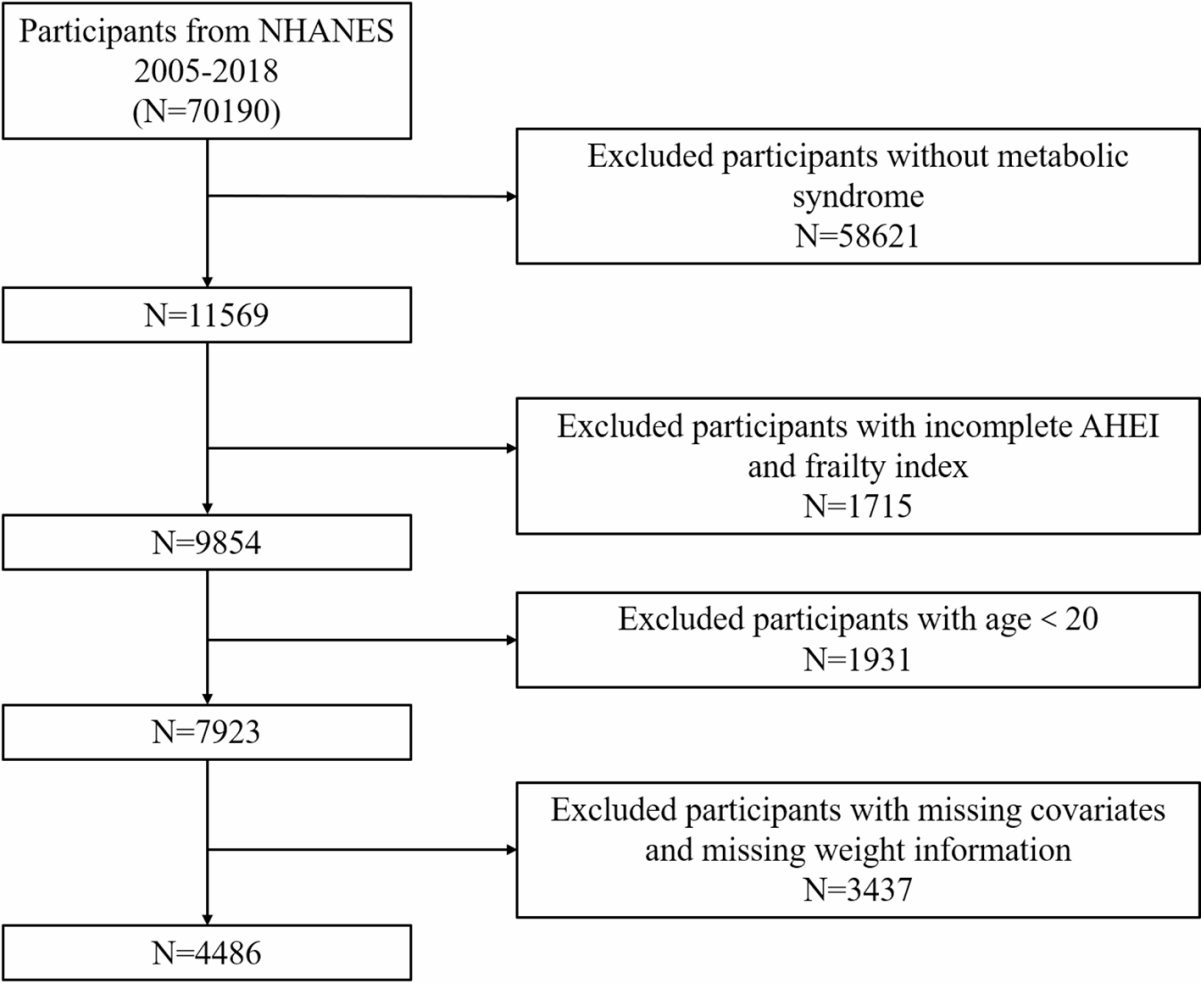
Flowchart for selection of participants for inclusion in the study. Abbreviation: AHEI, Alternative Healthy Eating Index

All participants included in the study needed to meet strict criteria, with the following specific exclusion criteria: (1) aged less than 20 years; (2) missing MetS diagnostic information; (3) lack of AHEI; (4) lack of weight information or weight = 0; (5) lack of other covariates (See Fig. 1).

### MetS diagnosis criteria

The diagnosis of MetS in the participating cohort followed the NCEP-ATPIII (National Cholesterol Education Program Adult Treatment Panel III) criteria [18]. Specifically, a participant was confirmed to be affected by MetS when the individual met ≥ 3 conditions listed below: (1) male and female high density lipoprotein cholesterol (HDL-C) levels lower than 1.04 and 1.29 mmol/L, respectively; (2) serum triglycerides (TG) ≥ 1.7 mmol/L; (3) male and female WC higher than 102 and 88 cm respectively; (4) FPG ≥ 6.1 mmol/L or currently receiving antidiabetic medication; (5) Blood pressure (BP) ≥ 130/85 mmHg.

### Frailty assessment

The Frailty Index (FI), calculated as the ratio between actual and total number of health deficits, was adopted for assessing the status of frailty. Herein, FI calculation considered a total of 49 health deficits, with the reference value range of 0–1. An FI value of 0 represents no frailty, while 1 denotes complete frailty. The FI value is therefore positively correlated with debilitation severity. In this way, we can convert continuous variables into categorical variables for further analysis. As for FI, we divided it into dichotomous variables. Participants with FI greater than or equal to 0.21 were categorized as being in a weakened state [19]. Table S1 details all the information.

### Calculation of AHEI-2010 scores

AHEI-2010, a parameter created by Harvard University investigators as a proxy indicator of dietary quality, can be utilized to predict the incidence of chronic disorders associated with dietary factors. The parameter contains 11 components, each of which is assigned a score of 0–10 [20]. The final AHEI-2010 value is the accumulated score for all the components. Higher scores represent healthier diets. When calculating the AHEI-2010 scores using the NHANES database, we adapted the calculation method based on the previous literature due to the lack of information on trans fatty acids in the NHANES database. Table S2 details all the algorithmic information.

**Fig. 2:**
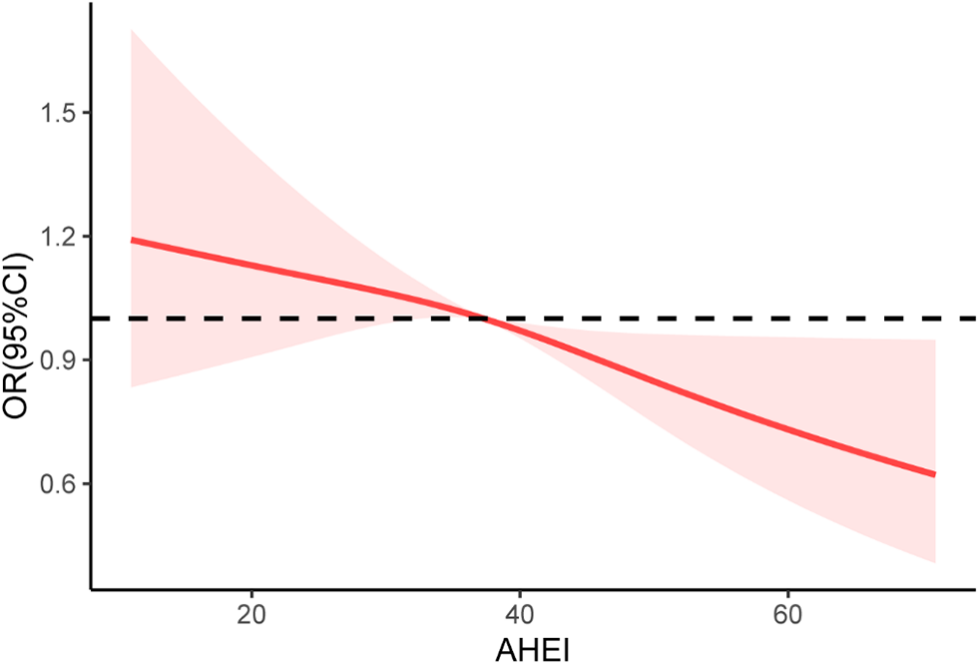
Associations between AHEI and frailty status in patients with metabolic syndrome. Abbreviation: AHEI, Alternative Healthy Eating Index

### Covariates

The questionnaire, demographic, laboratory and examination data of NHANES 2005–2018 were employed for variable extraction. Demographically relevant covariates involved age, gender, race, family poverty income ratio (PIR), education level, and marital status. Of these, gender was categorized as male/female. Marital status included living with partner, never married, married, separated, divorced and widowed. Education level involved lower than high school, high school, and higher than high school. PIR was categorized as low (PIR < 1.30), medium (1.30 ≤ PIR < 3.50) and high (≥ 3.50) with reference to previous studies [21, 22]. Laboratory data included serum albumin (Sal). According to the official NHANES document, the DcX800 method is a two-color digital endpoint method for measuring Sal concentration. The participants were also categorized into never, former, and current smokers according to their answers to the questionnaire.

### Statistical analysis

As per recommendations of NHANES, the data were analyzed while considering a complex sampling design and sampling weights to make this study more nationally representative. We used 0.21 as the threshold for FI to categorize the participant population into two broad categories of frailty and non-frailty status. Qualitative and continuous variables were respectively described with numbers (weighted percentages) and mean ± standard error (SE) and were comparatively analyzed through chi-square and Student’s t tests. For assessing associations between AHEI and frailty status, weighted multivariate logistic regression models were established, with findings displayed as odds ratio (OR) and 95% confidence interval (CI). Restricted cubic spline (RCS) based on model 3 was implemented for determination of dose-response AHEI-frailty relationship. Additionally, subgroup analyses and interaction test were carried out.

R (4.2.2) software was employed for all the aforementioned tests. The AHEI score was calculated using the “dietaryindex”package [23]. Two-tailed *P* < 0.05 was deemed to represent statistical significance for all the assays.

## Results

### Baseline participant information

The included cohort comprised 4486 MetS individuals with a mean age of 54.76 ± 0.34 years and a male proportion of 47.48% (2074 individuals). The highest percentage of race was Non-Hispanic White (72.88%), and marital status was predominantly married (60.21%). An FI value of 0.21 was set as the cutoff to divide the cohort into non-frailty and frailty groups. We found that 1623 participants were in a frailty status (FI ≥ 0.21). Table 1 summarizes significant differences between the non-frailty and frailty groups regarding the aforementioned demographic and clinical variables. Notably, the frailty group displayed higher age, higher female proportion, and lower AHEI scores compared with non-frailty MetS participants. In particular, participants in the frailty group had an AHEI score of 36.75 ± 0.38, whereas participants in the non-frailty group had a score of 38.29 ± 0.29.

**Table 1:** The baseline characteristics of the included participants, grouped according to whether or not they are in the frailty status. (weighted)

Variables	Total (*N* = 4486)	Non-Frailty (*N* = 2863)	Frailty (*N* = 1623)	*P*-value
**Age, y**	54.76(0.34)	52.65(0.42)	59.45(0.39)	< 0.0001
**Gender, n(%)**				< 0.0001
Male	2074(47.48)	1456(53.18)	618(34.84)	
Female	2412(52.52)	1407(46.82)	1005(65.16)	
**Race, n(%)**				< 0.0001
Mexican American	697(7.66)	501(8.54)	196(5.70)	
Other Hispanic	394(4.15)	258(4.09)	136(4.26)	
Non-Hispanic White	2220(72.88)	1417(74.14)	803(70.07)	
Non-Hispanic Black	851(9.72)	466(7.90)	385(13.78)	
Other Race	324(5.59)	221(5.33)	103(6.18)	
**Marital status, n(%)**				< 0.0001
Married	2540(60.21)	1715(62.65)	825(54.81)	
Widowed	500(8.95)	239(6.79)	261(13.75)	
Divorced	594(12.40)	345(11.47)	249(14.47)	
Separated	145(2.07)	78(1.54)	67(3.24)	
Never married	442(9.99)	299(10.81)	143(8.16)	
Living with partner	265(6.38)	187(6.74)	78(5.58)	
**Education level, n(%)**				< 0.0001
Below high school	1185(17.68)	662(15.07)	523(23.47)	
High school	1142(27.35)	712(26.13)	430(30.05)	
Above high school	2159(54.97)	1489(58.80)	670(46.48)	
**BMI, kg/m** ^**2**^	33.39(0.16)	32.89(0.18)	34.50(0.26)	< 0.0001
**PIR**	2.89(0.05)	3.14(0.05)	2.33(0.07)	< 0.0001
**Smoke, n(%)**				< 0.0001
Never	2234(49.08)	1522(52.60)	712(41.27)	
Former	1410(32.28)	869(31.18)	541(34.71)	
Current	842(18.64)	472(16.22)	370(24.02)	
**TG, mmol/L**	2.03(0.03)	2.02(0.03)	2.05(0.06)	0.67
**HDL-C, mmol/L**	1.20(0.01)	1.18(0.01)	1.23(0.02)	0.002
**FPG, mmol/L**	6.74(0.05)	6.54(0.05)	7.18(0.08)	< 0.0001
**WC, cm**	111.33(0.34)	110.35(0.40)	113.56(0.54)	< 0.0001
**Sal, g/L**	41.59(0.09)	42.08(0.08)	40.49(0.14)	< 0.0001
**AHEI**	37.81(0.24)	38.29(0.29)	36.75(0.38)	0.001
**Hypertension, n(%)**				< 0.0001
Yes	2814(59.48)	1500(51.09)	1314(78.09)	
No	1672(40.52)	1363(48.91)	309(21.91)	

Data are presented as weighted mean (SE) or numbers (weighted percentage)

Abbreviation: BMI, body mass index; PIR, poverty income ratio; TG, Triglyceride; HDL-C, High density lipoprotein cholesterol; AHEI, Alternative Healthy Eating Index; FPG, fasting plasma glucose; WC, Waist circumference; Sal, serum albumin

### Association between AHEI and frailty status

We assessed the AHEI-frailty relationship among the collected MetS cohort using weighted logistic regression models. See Table 2 for more detailed information. We established a total of 3 models. Model 1 was unadjusted. Age, race, and gender were adjusted for in Model 2, based on which further adjustments were made in Model 3 considering marital status, education level, smoking status, PIR, and Sal. The results revealed that the significant negative AHEI-frailty assocciation persisted for all these models when AHEI was used as a continuous variable (*P* < 0.05).

**Table 2:** The association between AHEI and frailty status among patients with metabolic syndrome (weighted)

Exposure	Model I	Model II	Model III
	OR (95%CI), *P* value	OR (95%CI), *P* value	OR (95%CI), *P* value
AHEI	0.99(0.979,0.995) 0.002	0.97(0.964,0.982) < 0.001	0.99(0.981,0.998) 0.022
AHEI (quartile)			
Q1	Ref	Ref	Ref
Q2	0.92(0.73,1.16) 0.49	0.78(0.60,1.02) 0.07	0.91(0.69,1.19) 0.48
Q3	0.82(0.65,1.04) 0.09	0.62(0.48,0.81) < 0.001	0.82(0.61,1.09) 0.16
Q4	0.64(0.49,0.84) 0.001	0.43(0.32,0.59) < 0.001	0.68(0.51,0.92) 0.01
*P* for trend	< 0.001	< 0.001	0.010

Model I: Non-adjusted; Model II Age, Gender, Race; Model III Age, Gender, Race, Marital status, Education level, PIR, smoke, Sal

Subsequently, categorizing all participants into tertile based on AHEI levels, we found that the highest AHEI quartile (Q4) of individuals possessed a significantly lower likelihood of frailty compared to participants with low AHEI scores when not adjusted for any covariates (OR = 0.64, 95% CI = 0.49–0.84, *P* = 0.001). Again, the correlation persisted following all covariates were adjusted for (OR = 0.68, 95% CI = 0.51–0.92, *P* = 0.01).

The RCS analysis of the participating MetS cohort did not find a nonlinear AHEI-frailty relationship (*P* for nonlinear = 0.505). See Fig. 2.

### Subgroup and interaction analyses

Table 3 displays the findings of subgroup and interaction analyses, which indicated that AHEI maintained a negative association with frailty status in all subgroups, while no significant interactions were detected between gender, age, race, smoking status, and PIR with AHEI (*P* > 0.05 for all interactions).

**Table 3:** Subgroup analysis for the association between AHEI and frailty status in patients with metabolic syndrome

	OR 95% CI	*P* value	*P* for interaction
**Age**			0.09
<60	1.00(0.99,1.01)	0.71	
≥60	0.99(0.98,1.00)	0.05	
**Gender**			0.54
Male	0.99(0.98,1.01)	0.43	
Female	0.99(0.974,0.998)	0.03	
**Race**			0.43
Mexican American	0.99(0.97,1.02)	0.47	
Other Hispanic	0.98(0.96,1.01)	0.24	
Non-Hispanic White	0.99(0.98,1.00)	0.12	
Non-Hispanic Black	0.99(0.98,1.01)	0.43	
Other Race	0.98(0.94,1.01)	0.12	
**Smoke**			0.76
Never	0.99(0.98,1.00)	0.19	
Former	0.99(0.97,1.00)	0.16	
Current	0.99(0.97,1.01)	0.24	
**PIR level**			0.96
<1.3	0.99(0.98,1.01)	0.39	
1.3–3.5	0.98(0.97,0.99)	0.002	
≥ 3.5	0.99(0.98,1.01)	0.45	

## Discussion

So far as we know, this research pioneers in linking AHEI to the chance that patients with MetS will become frail. Specifically, the current investigation explored the AHEI-frailty correlation, with frailty status reflected by FI. Our findings highlight a robust negative correlation between AHEI score and frailty development risk that persisted across all subgroups.

Diet may affect MetS risk through an individual’s general dietary patterns, consumption of particular foods, or specific nutrients, which may elevate or mitigate the risk of chronic disease. The AHEI assesses dietary quality and compliance with a healthy eating pattern, offering superior guidance for a nutritious diet to enhance health factor monitoring and increase predictive accuracy for chronic diseases [20]. Compared to the HEI, the AHEI focuses more on fat quality, promotes the consumption of nuts and legumes, and suggests that drinking alcohol in moderation contributes to good health [24]. The AHEI also recommends avoiding sugary drinks and fruit juices in the diet, and the higher the AHEI score, the healthier the participant’s dietary patterns and the lower the risk of chronic disease.

Several key features of MetS, including obesity, insulin resistance (IR), and hyperglycemia, are strongly associated with frailty [25]. A previous cross-sectional study found that people with MetS were about 50% more likely to be frail or pre-frail [26]. He et al. conducted an analysis of prospective cohorts from China and the United Kingdom, respectively, and found that metabolically unhealthy overweight/obesity and normal weight accelerated the progression of frailty [27]. The mechanism may be related to the imbalance of immune cells recruited from adipose tissue in MetS patients, which in turn induces systemic inflammation. The mechanism for this may be related to an imbalance of immune cells recruited from adipose tissue in patients with MetS, which in turn induces systemic inflammation [28]. In addition, systemic oxidative stress induced by IR and obesity may activate a downstream inflammatory cascade, and patients with MetS have elevated levels of various inflammatory markers, including C-reactive protein (CRP) and interleukin (IL)-6 [28].

It is now recognized that chronic systemic inflammation is one of the major determinants of the development of frailty [29]. An increasing amount of evidence supports the notion that nutrition can affect the equilibrium between pro- and anti-inflammatory cytokines and adipokines, indicating that chronic low-grade inflammation may potentially be influenced by dietary practices [30, 31]. Multiple epidemiological studies have established the correlation between diverse dietary elements and inflammation, as well as inflammatory markers. Previous studies have found that intake of phytonutrients reduces systemic inflammation, whereas intake of red meat and excessive dairy products may have the opposite effect [32]. Clinical studies conducted by Daniels et al. found that increased intake of fruits and vegetables increased serum levels of carotenoids and affected enzymes associated with the antioxidant properties of HDL [33]. A meta-analysis conducted by Khodarahmi et al. found that natural soy products reduced high-sensitivity CRP levels [34]. Another systematic evaluation provided evidence that plant-based dietary patterns are associated with lower levels of oxidative stress and inflammation [35]. A meta-analysis by Santos et al. found a positive correlation between saturated fatty acids (SFA) and ultrasensitive CRP [36]. A systematic evaluation by Rausch’s team found that marine-sourced omega-3 fatty acid (EPA + DHA) supplementation significantly reduced the leptin-lipocalin ratio (LAR) [37]. The health effects of alcohol vary according to the amount and pattern of alcohol consumption [38]. SSB intake has been shown to be positively associated with CRP levels [39]. SSB can also lead to high sugar intake, low satiety and obesity [40, 41].

In addition, diet can intervene in the balance of pro- and anti-inflammatory responses in the gut by influencing the gut microbiota. Some studies have found higher abundance of commensal bacteria such as *Roseburia*, *Faecalibacterium and Eubacterium spp* in the gut microbiota after consumption of fruits, vegetables, grains and red wine [42]. These bacteria can exert anti-inflammatory effects in the gut by fermenting fiber into short-chain fatty acids (SCFA). A high-fat diet enhances the permeability of intestinal epithelial cells and affects the interaction between the local intestine mucosal immune system and the intestinal microbiota, leading to an imbalance in microbiota composition [43]. This imbalance is characterized by an increase in the number of lipopolysaccharide (LPS)-producing Gram-negative bacteria, which in turn leads to persistent low-grade inflammation both locally and systemically [44].

From this, it can be seen that following the recommendations of the AHEI-2010 Dietary Scoring System, especially increasing the intake of fruits, vegetables, whole grains, and legumes, encouraging the consumption of healthy fats (including limiting trans fats and promoting polyunsaturated fats), decreasing sodium intake, drinking alcohol in moderation, and decreasing the intake of sugar-sweetened beverages, may reduce the risk of frailty in the MetS population.

Our research possesses multiple strengths. This study utilizes data from seven cycles of the NHANES database spanning 2005 to 2018, thereby guaranteeing a sufficiently high sample size for our analysis. The NHANES database employs a sophisticated multi-stage probability sampling technique, enhancing the generalizability of the results. Second, we adjusted for multiple confounding variables to verify the stability of the results.

Nonetheless, it is indisputable that our study possesses certain limitations. The cross-sectional design of the current investigation precluded the establishment of a causal link between AHEI and frailty status in the MetS population. Future large-scale prospective studies are needed to further confirm our current findings. Second, the populations included in this study were adult Americans, so the findings may not be fully applicable to other countries and regions, and more caution is needed when interpreting the results. In addition, the diet-related information in this study was obtained from the subjects’ recollections, which may result in unavoidable bias.

## Conclusion

This cross-sectional study utilizing NHANES data indicates that the AHEI is inversely correlated with the risk of frailty in individuals with MetS. Elevated AHEI scores would substantially diminish the likelihood of acquiring frailty. The results of this study hold significant significance for clinical practice and may direct doctors to concentrate on the dietary behaviors of patients with MetS. Compliance with a nutritious food regimen can significantly diminish the risk of frailty in individuals with MetS and alleviate the disease burden.

## Electronic supplementary material

Below is the link to the electronic supplementary material.

Supplementary Material 1

## Data Availability

No datasets were generated or analysed during the current study.
