# Supplementary material for: Correlation of alternative healthy eating index with risk of frailty among metabolic syndrome individuals: a cross-sectional study
Source: Aging Clin Exp Res. 2025 Mar 17;37(1):91. doi: 10.1007/s40520-025-02992-y (PMC11914318; doi:10.1007/s40520-025-02992-y)
Supplement: Supplementary file 1 — Supplementary Material 1 [file 40520_2025_2992_MOESM1_ESM.docx]

**Supplementary Material**

**Table S1**. Variables in the 49-Item frailty index and their respective scorings

| Variable | Scoring | | |
| --- | --- | --- | --- |
| Cognition | Yes=1, No=0 |  |  |
| 1. Experience confusion/memory problems | Difficulty=1, No Difficulty=0 |  |  |
| Dependence | Difficulty=1, No Difficulty=0 |  |  |
| 2. Managing money | Difficulty=1, No Difficulty=0 |  |  |
| 3. Stooping, crouching, kneeling | Difficulty=1, No Difficulty=0 |  |  |
| 4. Lifting or carrying | Difficulty=1, No Difficulty=0 |  |  |
| 5. House chore | Difficulty=1, No Difficulty=0 |  |  |
| 6. Preparing meals | Difficulty=1, No Difficulty=0 |  |  |
| 7. Standing up from armless chair | Difficulty=1, No Difficulty=0 |  |  |
| 8. Getting in and out of bed difficulty | Difficulty=1, No Difficulty=0 |  |  |
| 9. Using fork, knife, drinking from cup | Difficulty=1, No Difficulty=0 |  |  |
| 10. Dressing yourself | Difficulty=1, No Difficulty=0 |  |  |
| 11. Standing for long periods difficulty | Difficulty=1, No Difficulty=0 |  |  |
| 12. Grasp/holding small objects | Difficulty=1, No Difficulty=0 |  |  |
| 13. Attending social event | Difficulty=1, No Difficulty=0 |  |  |
| 14. Push or pull large objects | Difficulty=1, No Difficulty=0 |  |  |
| 15. Walking for a quarter mile difficulty | Difficulty=1, No Difficulty=0 |  |  |
| 16. Walking up 10 steps difficulty | Difficulty=1, No Difficulty=0 |  |  |
| Depressive Symptoms |  |  |  |
| 17. Have little interest in doing things | Nearly every day=1, More than half the days=0.66, Several days=0.33, Not at all=0 |  |  |
| 18. Feeling down, depressed, or hopeless | Nearly every day=1, More than half the days=0.66, Several days=0.33, Not at all=0 |  |  |
| 19. Trouble sleeping or sleeping too much | Nearly every day=1, More than half the days=0.66, Several days=0.33, Not at all=0 |  |  |
| 20. Feeling tired or having little energ | Nearly every day=1, More than half the days=0.66, Several days=0.33, Not at all=0 |  |  |
| 21. Poor appetite or overeating | Nearly every day=1, More than half the days=0.66, Several days=0.33, Not at all=0 |  |  |
| 22. Feeling bad about yourself | Nearly every day=1, More than half the days=0.66, Several days=0.33, Not at all=0 |  |  |
| 23. Trouble concentrating on things | Nearly every day=1, More than half the days=0.66, Several days=0.33, Not at all=0 |  |  |
| Comorbidities |  |  |  |
| 24. Arthritis | Yes=1, Suspect=0.5, No=0 |  |  |
| 25. Thyroid problems | Yes=1, Suspect=0.5, No=0 |  |  |
| 26. Chronic bronchitis | Yes=1, Suspect=0.5, No=0 |  |  |
| 27. Cancer | Yes=1, Suspect=0.5, No=0 |  |  |
| 28. Congestive heart failure | Yes=1, Suspect=0.5, No=0 |  |  |
| 29. Coronary heart disease | Yes=1, Suspect=0.5, No=0 |  |  |
| 30. Angina | Yes=1, Suspect=0.5, No=0 |  |  |
| 31. Heart attack | Yes=1, Suspect=0.5, No=0 |  |  |
| 32. Stroke | Yes=1, Suspect=0.5, No=0 |  |  |
| 33. Blood pressure | Yes=1, Suspect=0.5, No=0 |  |  |
| 34. Diabetes | Yes=1, Suspect=0.5, No=0 |  |  |
| 35. weak/failing kidneys | Yes=1, Suspect=0.5, No=0 |  |  |
| 36. Urinary Leakage | Yes=1, Suspect=0.5, No=0 |  |  |
| Hospital Utilization and Access to Care |  |  |  |
| 37. Self-rated health | Fair, poor=1, Excellent, Very good, good=0 |  |  |
| 38. Health now compared with 1 year ago | Worse=1, About the same, better=0 |  |  |
| 39. Overnight hospital patient in past year | Yes=1, No=0 |  |  |
| 40. Frequency of health care use during past year | None=0, 1-5=0.5, More than 5=1 |  |  |
| 41. Number of prescribed medications | None=0, 1-4=0.5, 5 and more=1 |  |  |
| Physical Performance and Anthropometry |  |  |  |
| 42. Body mass index | <18.5, ≥30=1 |  |  |
|  | 25-<30=0.5 |  |  |
|  | 18.5-25=0 |  |  |
| 43. Handgrip strength | MALE: |  | FAMELE: |
|  | For BMI≤24，GS≤29 |  | For BMI≤23，GS≤17 |
|  | For BMI24.1-28, GS≤30 |  | For BMI23.1-26,GS≤17.3 |
|  | For BMI＞28, GS≤32=1 |  | For BMI26.1-29,GS＜18 |
|  |  |  | For BMI＞29,GS≤21=1 |
| Laboratory Values |  |  |  |
| 44. Glycohemoglobin (%) | 0%-5.7%=0, >5.7%=1 |  |  |
| 45. Red blood cell count (million cells/mL) | M: 4.7-6.1=0, Other=1 |  | F: 4.2-5.4=0, Other=1 |
| 46. Hemoglobin (g/dL) | M: 13.5-18=0, Other=1 |  | F: 12-16=0, Other=1 |
| 47. Red cell distribution width (%) | 11.6-14.6=0, Other=1 |  |  |
| 48. Lymphocyte percent (%) | 20-40=0,Other=1 |  |  |
| 49. Segmented neutrophils percent (%) | 40-80=0, Other=1 |  | |

**TableS2 The calculation methods of AHEI**

| **Component** | **Description** | **Units** | **Minimum score: 0** | **Maximum score: 10** |  |
| --- | --- | --- | --- | --- | --- |
| Vegetables | All vegetable except potatoes and legume | servings/day (0.5 c of vege; 1 cup of green leafy (1 cup = 236.59 g) | 0 | ≥5 |  |
| Whole Fruit | All whole fruits and no fruit juice | servings/day (0.5 c of berries; 1 cup=236.59 g; 1 med fruit (1 cup = 236.59 g) | 0 | ≥4 |  |
| Whole grain | Whole grains in whole grain products | grams/day | 0 | 75 (female),  90 (male) |  |
| Nuts/Legumes/vege protein | Nuts, legumes, and vegetable protein (e.g., tofu) | servings/day = 1 srv=1oz (28.35 g) of nuts and legume or 1 TBLSP peanut butter (15 mL), 1 cup legume = 4 oz | 0 | ≥1 |  |
| Long chain (omega-3) fats (EPA+DHA) | N/A | mg/day ( oz. = 28.35 g) | 0 | 250 |  |
| PUFA | N/A | % of energy | ≤2 | ≥10 |  |
| Sugar-Sweet Bevs (+fruit juice) | Intake of sugar-sweetened beverages, including soda and fruit drinks, but no 100% fruit juice | servings/day = 1 ser= 8oz (1 oz. = 28.35 g) | ≥1 | 0 |  |
| Red/processed meat | Beef, pork, lamb, goat, veal, sausages, bacon, salami, ham, hot dog, deli meat, organ meat | servings/day; 1 srv= 4 oz. unprocessed meat; 1.5 oz. processed meat (1 oz. = 28.35 g) | ≥1.5 | 0 |  |
| *trans* Fat |  |  |  |  | trans fat is not calculated for NHANES given that NHANES does not include it in the nutrition datasets and trans fat concentration in foods is changing every year since 2005 (reference 2) |
| Sodium | N/A | mg/day per 2000 kcal | Highest decile | Lowest decile |  |
| Alcohol | Wine, beer, "light" beer, liquor | drink/day (12 oz. beer; 5 oz. wine; 1.5 oz. spirits) 1 oz = 28.35 g | Women: > 2.5,  Men: > 3.5 | Women: 0.5-1.5,  Men: 0.5-2.0 |  |
|  |  | **Total possible score** | **0** | **100** |  |
